# A Rare Case of Darier Disease Flaring Up During Pregnancy

**DOI:** 10.7759/cureus.72176

**Published:** 2024-10-23

**Authors:** Vidya Gaikwad, Jay Patel, Himali Hatwar

**Affiliations:** 1 Obstetrics and Gynaecology, Dr. D. Y. Patil Medical College, Hospital and Research Centre, Dr. D. Y. Patil Vidyapeeth (Deemed to be University), Pune, IND

**Keywords:** autosomal dominant genetic disorder, darier’s disease, flare-up, normal spontaneous vaginal delivery (nsvd), pregnancy

## Abstract

Darier disease (DD), which was also known as keratosis follicularis or Darier-White disease, is an autosomal dominant hereditary illness. The illness typically first manifests in adolescence and progresses as a woman reaches reproductive age. Research on the potential effects of DD on the skin and fetus, especially when it affects the groin and vulva, is insufficient. We report a unique case of multiple folliculitis encompassing the skin of the axilla, breasts, belly, and groin during pregnancy. This condition was associated with recurrent episodes of chronic DD that flared up during each pregnancy and was safely delivered vaginally.

## Introduction

With considerable geographical and penetrance variability, Darier disease (DD) is an uncommon autosomal dominant genetic condition that affects roughly one in 30,000 persons [1-3]. Both genders are equally affected by the illness, which is caused by a mutation in the ATP2A2 gene [1,4]. Mutation in the ATP2A2 gene, which codes for the calcium (Ca²+) ATPase pumps (SERCA2) of the endoplasmic reticulum. By lowering Ca²+, this mutation results in acantholysis [5]. The sickness will coexist with a woman's reproductive years since it frequently first appears in adolescence [6]. The hallmarks of DD include dyskeratosis and suprabasal acantholysis, which manifest clinically as warty plaques and papules in seborrheic and flexural areas. It smells bad, is deformative, and frequently causes pruritus [6]. It usually affects the neck, forehead, groin, nails, chest, and back [1,2].

Sweat, heat, UV rays, and lithium carbonate are exacerbating factors. Along with itching and malodor, the most common secondary infections that flares may present are *Staphylococcus aureus*, herpes simplex virus, and human papillomavirus [5]. Previous research has confirmed the progression and amelioration of the condition throughout pregnancy, as well as secondary infections caused by bacteria and viruses [7]. While there are reports of flare-ups associated with menstruation and pregnancy, it is thought that these are less common because high estrogen environments tend to make Darier less active [8,9]. Additionally, there is a paucity of literature regarding the potential effects of DD during pregnancy, especially when it affects the abdomen and groin [10], which makes our case unique.

## Case presentation

**Figure 1:**
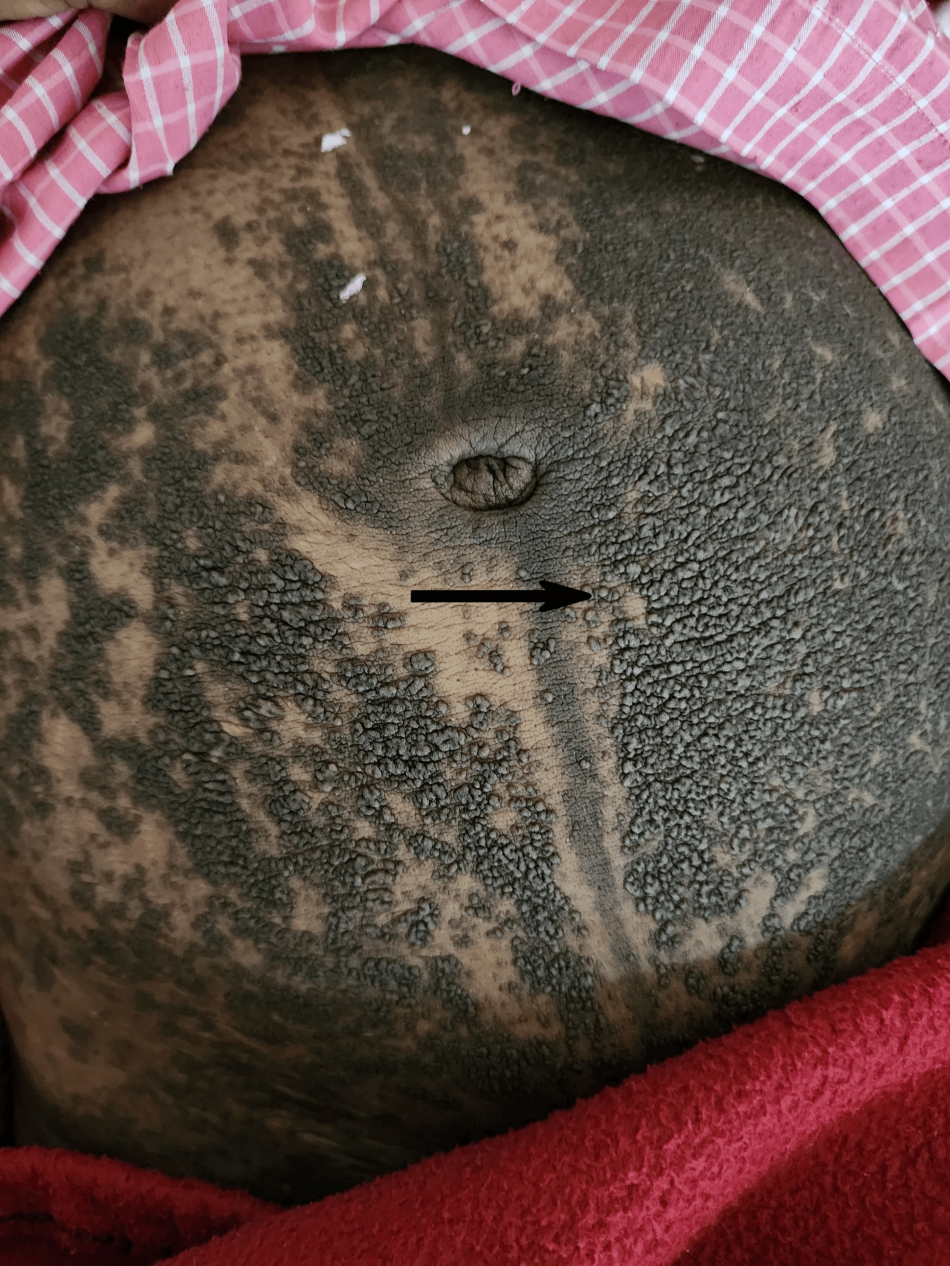
Skin lesions over the abdomen

**Figure 2:**
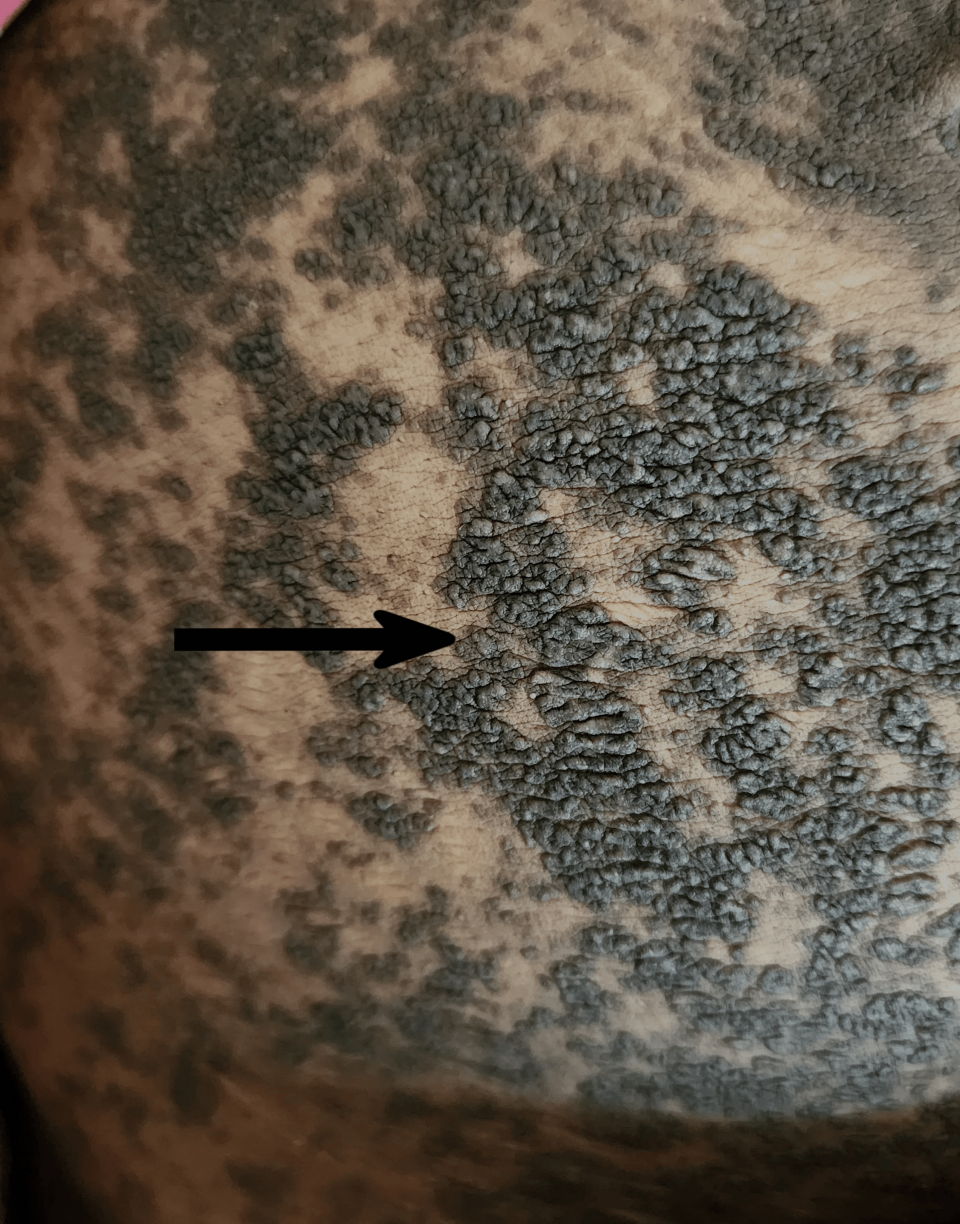
Skin lesions near the chest and below the axilla

A 35-year-old female with seventh gravida and fourth para came to the labor room with complaints of labor pain for one day. She had four previous vaginal deliveries; out of them, one baby died in utero at the seventh month of gestation. She also had a history of two spontaneous abortions at around the second month of gestation. She had her first antenatal care (ANC) checkup at the seventh month of gestation in this pregnancy. The patient was unable to recall the date of the last menstrual period. By the second trimester scan, she had a pregnancy of 40+6 weeks. She did not receive any antenatal vaccination or supplementation in pregnancy until delivery. On examination, she had mild contractions lasting for 10 to 20 seconds every 10 minutes. Per-vaginal examination showed 2 cm cervical dilatation with 30-40% effacement and head at station -1. On general examination, the patient revealed brownish, greasy, and malodorous warty lesions, seborrheic in flexural regions of the axilla, neck, and submammary area. She had such lesions for the last 10-11 years with itching, sometimes otherwise asymptomatic, for which she received irregular treatment with topical corticosteroids. These lesions flare up with every pregnancy and show remission three to four months after delivery. Lesions were extended over all abdomen, groin, and arms (Figure 1 and Figure 2).

Routine antenatal investigations were sent on admission, including a complete blood count and routine urine microscopy (Table 1 and Table 2). Dermatologist consultation was done to assess the current state of skin lesions, to manage any exacerbations, and to advise on safe topical or systemic treatments that can be used during labor, if necessary. 

**Table 1:** Blood investigations

Blood Parameters	Reference Range	Patient Value
Hemoglobin	11.6-15.0 g/dL	11.40
Total leucocytes (WBC) count	4000-10000/µL	9,800
Platelet count	150000-410000 /µL	111,000
Total bilirubin	0.22-1.20 mg/dL	0.62
Aspartate aminotransferase	8-43 U/Lt	33
Alanine aminotransferase	7-45 U/Lt	16
Alkaline phosphatase	35-104 U/Lt	353
Plasma glucose, random	<200 mg/dL	74
Uric acid	2.70-6.10 mg/dL	4.40
Urea	17-49 mg/dL	18
Creatinine	0.6-1.2 mg/dL	0.57
Thyroid-stimulating hormone (ultrasensitive)	0.35-4.94 µIU/mL	4.45
Prothrombin time/international normalized ratio	11.63/1.00	10.30/0.87

**Table 2:** Urine routine microscopy HPF, high-power field

Urine Routine Examination	Reference Interval	Result
Appearance	-	Clear
Color	-	Pale yellow
pH	4.6-8.0	7.5
Glucose	Absent	Absent
Acetone	Absent	Absent
RBCs	0-2 per HPF	6-8
Pus cells	0-5 per HPF	1-2

Thus, the diagnosis was made "A 35-year-old female, G7P4L3D1A2, with previous vaginal deliveries and 40+6 weeks of pregnancy with Darier disease in latent labor." She progressed well and delivered vaginally on the same day of admission. Delivery was uneventful, and the baby was shifted to the mother's side. A thorough dermatological evaluation was done postpartum to assess the current status of her DD and to confirm the diagnosis, given its recurrence during pregnancy and its effect on neonates. 

The patient received routine postnatal care. They were given a five-day course of Tablet Augmentin 625 mg, proton pump inhibitor (PPI) antacids, and non-steroidal anti-inflammatory drugs (NSAIDs). Additionally, a dermatologist prescribed a urea-based moisturizing cream for 15 days for symptomatic skin relief. The mother and baby were discharged on the fifth day after delivery based on the advice of the obstetrician and pediatrician. The patient was instructed to follow up with an obstetrician and a dermatologist after two weeks.

## Discussion

This is one of the rare cases of DD complicating pregnancy that has been reported. Pregnancy and DD have been the subject of a few articles [7,9,11,12]. Due to the autosomal dominant pattern of inheritance, which affects 50% of kids, DD is considered an obstetric condition. Therefore, at a preconception appointment, couples should be offered genetic counseling or referred to a high-risk obstetrician early in the pregnancy for counseling. Since the 1980s, prenatal diagnosis has been feasible [13]. Due to the disease's varied penetrance, genotype-phenotype correlation studies have shown unsatisfactory results, and it is impossible to anticipate the severity of the condition in an affected individual's kids, making prenatal counseling challenging in DD [4]. DD has a broad range of disease phenotypes, with many mild cases being untreated and severe cases having a significant negative influence on quality of life. In most cases, oral and/or topical retinoids are used to treat severe illness. Ninety percent of individuals respond clinically well to these medications. Nevertheless, they should not be used when pregnant, as they are teratogenic. Although they are not very effective, topical steroids can be used safely during pregnancy [2,6].

The current case study demonstrates how DD can lead to pregnancy complications when it affects the lower abdomen, necessitating a Pfannenstiel incision for a cesarean section delivery, or when it involves the groin, vulva, or perineum, where skin elasticity is crucial for atraumatic vaginal birth. If there is extensive back involvement that prevents the safe administration of regional anesthesia, it may also have an impact on delivery [12]. Acantholysis, which causes suprabasal clefting with papillomatosis and dyskeratosis, is a feature of DD [7,14]. The desmosomal plaque has been proposed as the main target of the chromosomal mutation [14]. A thinned skin barrier caused by histological alterations makes the skin more prone to infection. 

Obstetric complications arise from the existence of superimposed infections, particularly with regard to group B *Streptococcus*, which can cause severe newborn sepsis and even mortality. If a skin illness affects the breast, breastfeeding may also be impacted. There have been reports of neuropsychiatric correlations with DD. The majority of the connections that have been observed involve mental problems, which impact 50% of patients. Depression coupled with suicidal thoughts and attempts are frequent manifestations of these diseases [15]. It has previously been reported that DD improves when a woman is pregnant or uses oral contraceptives [9]. A 58-year-old lady who had an exacerbation of DD during each of her six pregnancies has also been documented by Spouge JD et al. [11]. Research on how a herpes or vaccinia virus skin infection during pregnancy can exacerbate DD has been conducted by Toole JWP et al. and Carney JF et al. [16,17]. There are not any documented instances of DD worsening during pregnancy with vegetating lesions and no subsequent infection, either.

## Conclusions

While the cause of DD is widely understood, nothing is known about the effects of genodermatosis on gynecology and pregnancy. Skin lesions in the abdomen and groin can complicate cesarean sections and affect the normal skin elasticity observed after vaginal delivery. This succinct case study aims to give practicing obstetricians and dermatologists some insights for future research on the proper care of pregnant women with DD.
